# Identification and validation of programmed cell death related biomarkers for the treatment and prevention COVID-19

**DOI:** 10.1080/07853890.2025.2492830

**Published:** 2025-04-29

**Authors:** Jie Yang, YaoXi Tan, Xing Liu

**Affiliations:** ^a^ Department of Infectious Diseases, Affiliated hospital of Jiangnan University, Wuxi, Jiangsu, China; ^b^ Department of Emergency, Affiliated Wuxi Fifth Hospital of Jiangnan University, Wuxi, Jiangsu, China

**Keywords:** Coronavirus disease 2019, programmed cell death, clinical features, immune microenvironment, subtypes classification

## Abstract

**Purpose:**

Programmed cell death (PCD) plays a key role in the progression of coronavirus disease 2019 (COVID-19). However, PCD-relevant biomarkers have not been fully discovered. The aim of this study was to explore the PCD-relevant biomarkers for the treatment and prevention of COVID-19.

**Methods:**

Bioinformatic analyses were performed to explore the clinical relevant PCD genes with differential expression (DE) in COVID-19 compared with matched controls. PPI network was used for hub genes screening and machine learning methods were employed for filtering feature genes. The biomarker genes were screened by Venn diagram. The correlations between biomarkers with clinical features and immune microenvironment were further explored. Biomarker validation was performed in clinical samples by real-time reverse transcriptase-polymerase chain reaction (RT-qPCR).

**Results:**

In total, 118 clinically relevant and PCD associated differential expressed genes (DEGs) were screened, which were mainly related with apoptosis related pathways, among which six biomarkers (Cyclin B1 (CCNB1), cyclin-dependent kinase 1 (CDK1), interferon regulatory factor 4 (IRF4), lipoteichoic acid (LTA), matrix metallopeptidase 9 (MMP9) and Oncostatin M (OSM)) were identified. The excellent or good diagnostic performance of biomarkers was determined by receiver operating characteristic (ROC) curve analysis. The biomarkers showed diverse correlations with clinical indicators, such as age, sex and Intensive Care Unit (ICU) admission. Total 14 types of immune cells exerted differential infiltration between COVID-19 and controls. Biomarkers were correlated with immune cells at varying levels. COVID-19 was classified in three clusters, which showed differential expression of biomarker genes and significant associations with clinical information, such as sex, age and ICU admission. The DEGs of biomarkers were determined in COVID-19 patients relative to controls.

**Conclusion:**

The six biomarkers (CCNB1, CDK1, IRF4, LTA, MMP9 and OSM) can be served as the biomarkers for the treatment and prevention of COVID-19.

## Introduction

The coronavirus disease 2019 (COVID-19) is a severe respiratory disease, which is stirred by a novel coronavirus. COVID-19 can cause various clinical presentations, from mild respiratory symptoms to organ damage, and even death [1]. Since its outbreak in 2019, COVID-19 has affected cases from over 200 countries, leading to 200,000 deaths at least [2]. COVID-19 as a global health concern is characterized by rapid spread through human-to-human transmission even among the asymptomatic [3], associated with severe and deadly complications. Effective biomarkers would help to understand the pathogenesis and facilitate prompt prevention. Thus, the identification of reliable biomarkers able to stratify patients with high risk is in urgent need.

Programmed cell death (PCD) plays a key role in the clearance of superfluous and damaged cells in various organ systems. The dysregulation of PCD is associated with the development and progression of various diseases, including cancer and pulmonary diseases [4]. It has been also indicated that the PCD pathways are involved in the immunity of host against bacterial and viral infections [5]. Emerging evidence has suggested PCD mediates the pathogenesis of COVID-19 by implicating in the inflammation and tissue damage [6,7]. For example, neutrophil extracellular trap (NET) formation (NETosis), as a form of PCD, is increased in the process of COVID-19 and contributes to the severity of disease, which has been served as a promising therapeutic option for COVID-19 [8]. Although the critical role of PCD has been determined in COVID-19, the biomarkers related with PCD for the diagnosis and prevention of COVID-19 are rarely discovered.

Therefore, in this study, three gene expression datasets related with COVID-19 were downloaded from Gene Expression Omnibus (GEO) database. The differential expression and weighted gene co-expression network analysis (WGCNA) analyses were performed to screen the clinically relevant PCD genes with differential expression between COVID-19 and matched controls. Further, the machine learning methods were employed to mine feature genes and protein–protein interaction (PPI) network was constructed to screen hub genes. We attempted to identify and validate the biomarkers of COVID-19, contributing to the understanding of pathogenesis of COVID-19 and preventing the severe complications.

## Methods

### Data source and processing

Three COVID-19-related datasets were downloaded from GEO database. The inclusion criteria are as follows: (1) Independent expression profile of COVID-19; (2) Data sets containing COVID-19 and matched Controls grouping information; (3) Include as large a sample size as possible, with a total sample size of at least 50 or more; (4) Test specimens from the whole blood dataset of Homo sapiens in the human body. GSE171110 gene expression profile was generated based on GPL16791 Illumina HiSeq 2500 platform, which contained whole blood samples of 44 COVID-19 patients and 10 matched controls. GSE157103 expression profile was produced from 100 blood samples from COVID-19 patients, and 26 from matched controls. GSE152641 expression dataset contained 62 whole blood samples from COVID-19 patients and 24 from matched controls. GSE157103 and GSE152641 datasets were produced based on GPL24676 Illumina NovaSeq 6000 (Homo sapiens) platform. The database is publicly available.

The raw datasets and annotation files were downloaded based on the sequencing platform. The probes were mapped to gene symbols and the probes without gene symbols were removed. If multiple probes were assigned to a specific gene, the mean expression value was calculated. Finally, the gene expression matrix was obtained for subsequent analysis.

### Differentially expressed genes (DEGs) analysis

Based on GSE171110 dataset, the genes differentially expressed in COVID-19 patients compared with controls were analysed by limma package Version 3.58.1 (https://bioconductor.org/packages/release/bioc/html/limma.html) in R. *P* values were corrected by Benjamini and Hochberg method. Genes with adjusted *p* value <0.05 and |log2FC (fold change) | ≥ 1 were set as the cutoff value for DEGs.

### WGCNA

To explore the clinically relevant modules and genes in COVID-19, WGCNA package [9] in R (version 1.72-5) (https://horvath.genetics.ucla.edu/html/CoexpressionNetwork/Rpackages/WGCNA/) was employed. For network construction, the optimal power (β) was determined based mean connectivity and scale independence value. Modules with co-expressed genes were divided with the cutoff value of module gene number ≥ 50. The correlations between modules and clinical traits were analysed in GSE157103 training dataset.

### Clinically PCD-associated genes

The PCD-associated genes were retrieved from previous studies [10,11]. Venn diagram analysis was performed to identify the overlapped genes with DEGs, WGCNA module genes and PCD-associated genes. The intersecting genes were defined as key genes and used for the following studies.

### Function enrichment analysis

Gene Ontology (GO) and Kyoto Encyclopedia of Genes and Genomes (KEGG) enrichment analyses were performed using R ‘clusterProfiler’ package [12] version 4.10.1 (http://bioconductor.org/packages/release/bioc/html/clusterProfiler.html). Based on GO database, genes were annotated into three categories, including MF (molecular function), cellular component (CC) and biological process (BP). Multiple testing was achieved by Benjamini and Hochberg method. The adj. *P*. Value <0.05 was set as the cut-off value. The top 10 significant GO function and pathways were visualized.

### Protein–protein interaction (PPI) network

The key genes were mapped to protein interaction pairs by using STRING database version 11.0 (http://string-db.org/). A PPI network was created with the application of Cytoscape software [13] version 3.8.2 (https://cytoscape.org/). CytoHubba plugin in Cytoscape was employed for searching hub genes based on five topology analysis algorithms (Maximal Clique Centrality (MCC), Maximum Neighbourhood Component (MNC), Edge Percolated Component (EPC) and Degree). The top 65 hub genes were filtered.

### Feature gene screening by machine learning methods

Three machine learning methods were adapted to screen feature genes related with COVID-19, including Least Absolute Shrinkage and Selection Operator (LASSO), random forest (RF), and Support Vector Machine-Recursive Feature Elimination (SVM-RFE). LASSO logistic regression [14], RF [15] and SVM-RFE [16] were widely used for characteristic gene screening. In the present study, LASSO logistic regression was implemented by R package ‘glmnet’ [17] version 4.1-8. LASSO model was trained by 10-fold cross-validation. RF model was conducted by using ‘randomForest’ [18] version 4.7–1.1. MeanDecreaseGini > 1 was used as the variable importance measure and feature genes were screened by 10-fold cross validation. SVM-RF algorithm was performed by using ‘e1071’ package [19] version 1.7–14. The feature genes identified by three machine learning methods were overlapped and the overlaps were considered as candidate biomarkers for COVID-19.

### Diagnostic performance and clinical significance of biomarker genes

The diagnostic performance of the biomarkers was evaluated by receiver operating characteristic (ROC) curve analysis with the application of pROC version1.18.5 [20] (http://expasy.org/tools/pROC/) in training and validation datasets. Correlation analysis was performed for feature genes and clinical indicators.

### Drug prediction for feature genes

Drug Signatures Database (DSigDB) is a drug signature database for linking drug/component with target genes [21]. The candidate drugs targeting feature genes were predicted based on DSigDB database.

### Nomogram model construction

To validate the diagnostic value of feature genes, the nomogram model was constructed by integrating gene signatures and clinical factors. The nomogram construction was performed using rms package [22] version 6.8-0 in R. The predictive value and stability of nomogram was evaluated by calibration curve. Decision curve analysis and clinical impact curve analysis were employed to assess the clinical utilization of nomogram in COVID-19 diagnosis.

### Immune cell infiltration analysis

The infiltration proportion of 28 types of immune cells were estimated based on gene expression values in training dataset, which was achieved by using single-sample gene set enrichment analysis (ssGSEA) algorithm [23]. The differences of immune cell percentage in COVID-19 vs. control were analysed by Wilcoxon test. The ggplot2 package version 3.5.0 (https://github.com/tidyverse/ggplot2) was employed for the correlation analysis between feature genes and immune cells.

### COVID-19 subtypes analysis and correlation between clinical information

COVID-19 samples were subjected to hierarchical clustering analysis using ‘ConsensusClusterPlus’ package version 1.66.0 [24] in R. The optimal number of clusters was identified based on K-mean algorithm. Principal component analysis (PCA) curve was applied for verifying the accuracy of the choice of K value. The correlation between clusters and clinical indicators were analysed.

### The immune microenvironment comparison between different clusters

The immune score, stromal score and ESTIMATE score of each cluster were calculated using ESTIMATE package [25] in R. The proportion of immune cells were evaluated based on ssGSEA algorithm. Differences of immune microenvironment between clusters were analysed based on Wilcoxon test.

### Real-time reverse transcriptase-polymerase chain reaction (RT-qPCR)

The blood samples from six COVID-19 patients and six paired healthy controls were collected. This study was conducted in accordance with the Declaration of Helsinki and received ethical approval from Affiliated Wuxi Fifth Hospital of Jiangnan University. Written consent was obtained from all participants. RNA was prepared based on Trizol method. cDNA synthesis was achieved by cDNA synthesis kit (mlbio, Shanghai, China) as per the manufactory’s instruction. Gene amplification was performed under Quantstudio7 Flex real-time PCR system (ThermoFisher). The primers for Cyclin B1 (CCNB1), cyclin-dependent kinase 1 (CDK1), interferon regulatory factor 4 (IRF4), lipoteichoic acid (LTA), matrix metallopeptidase 9 (MMP9) and Oncostatin M (OSM) were listed in Table 1. With GAPDH as the reference gene, the gene expression was semi-quantitatively analysed based on the previous method [26].

**Table 1.** Primer sequence for RT-qPCR analysis.

Gene symbol	Forward (5’-3’)	Reverse (5’-3’)
CCNB1	CTTAGACAAATTCTGAACTAGTGTACA	ATTCTTGACAACGGTGAAT
CDK1	GCCTAAAATGGCCTGAAAGC	CTCCTGCCATGTCCCTCTAG
IRF4	AGCGCATTTCAGTAAATGTAAACACAT	TCTTGTGTTCTGTAGACTGCCATCA
LTA	TCCAGGATCCGAGGTCCTGGAGGCTCTTTC	CCAGGGATCCCAGCTATCCACCCACACAGA
MMP9	CCTGGAGACCTGAGAACCAATC	GATTTCGACTCTCCACGCATC
OSM	GCAGACTCCTGGACCCTAT	TCAGCCGTGTCTGAGTTGTC
GAPDH	GGCAGTFATGGCATGGACTGT	CCTTCATTGACCTCAACTACA

### Statistical analysis

R 4.3.3, SPSS 22.0 and GraphPad Prism 7.0 software were used for data processing, statistical analysis and drawing. Measurement data followed normal distribution after normal test analysis and were expressed as mean ± standard deviation. The correlation between two continuous variables was assessed by Pearson’s correlation coefficient. The chi-square test was used to compare categorical variables, and the Wilcoxon test or t-test was used to compare continuous variables. ROC curve analysis of pROC (version1.18.5) was used to evaluate the diagnostic performance of biomarkers. Differences between the two groups of samples were estimated by *t*-test. All statistical tests were two-sided, and *P* values of less than 0.05 were considered statistically significant.

## Results

### Identification of the DEGs

**Figure 1.**
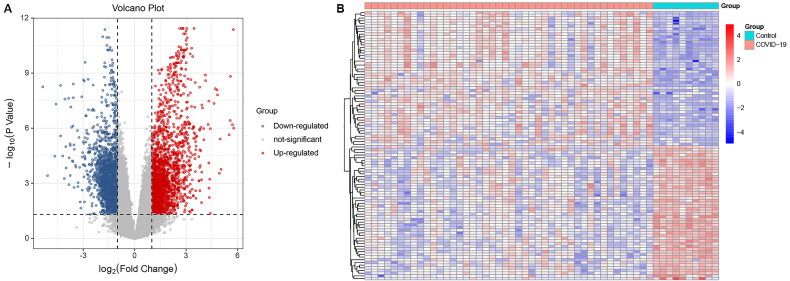
Differential expressed genes (DEGs) in COVID-19 patients compared with healthy controls. A, Volcano plot of DEGs. B, heatmap of DEGs.

Based on GSE171110 dataset, 3803 DEGs were identified in the blood samples of COVID-19 patients compared with matched controls. As shown in Figure 1(A), 2020 genes are upregulated, while 1783 genes are downregulated. The heatmap of top 50 upregulated and downregulated genes shows that the expression profiles of DEGs can clearly distinguish COVID-19 and control samples (Figure 1(B)).

### Clinically relevant modules identified by WGCNA

**Figure 2.**
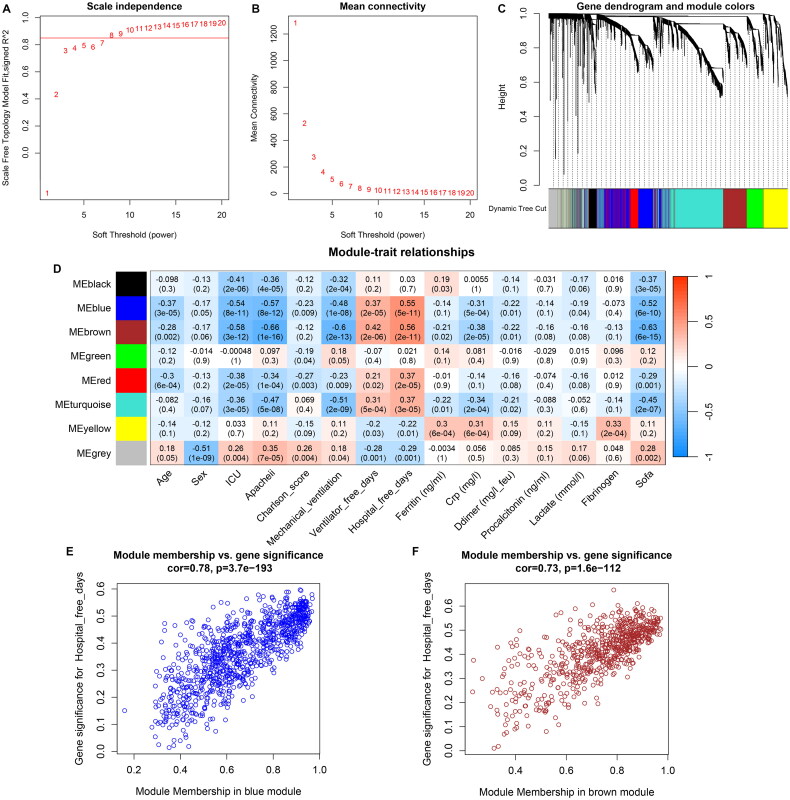
Clinically relevant genes analysed by WGCNA. A, B Graphs of scale independence, mean connectivity and scale-free topology, the appropriate soft-power was 8. C, Cluster dendrogram of the coexpression network modules. D, Correlation between modules with clinical traits. E, Correlation between blue module genes with free hospital days. F, Correlation between brown module genes with free hospital days.

All the genes were ranked in descending order based on median absolute deviation (MAD). Total 5000 genes with top MAD were subjected to co-expression network construction. To fit the scale-free feature, power of β = 8 was selected for network generation (Figure 2(A,B)). Genes in co-expression network were clustered into eight distinct modules represented with different colours (Figure 2(C)). The associations between modules and clinical traits were further explored. The heat map of the correlation between modules and traits is visualized in Figure 2(D). Based on the correlation coefficients, the blue (Cor = 0.55, p = 5e-11) and brown modules (Cor = 0.56, p = 2e-11) showed the strongest association with the hospital-free days. Thus, 940 genes in blue module and 670 genes in brown module were used for subsequent analyses. The link strength between gene signature and module membership in blue and brown modules was visualized in scatter plot (Figure 2(E,F)).

### Clinically PCD-associated genes identification and functional enrichment analysis

**Figure 3.**
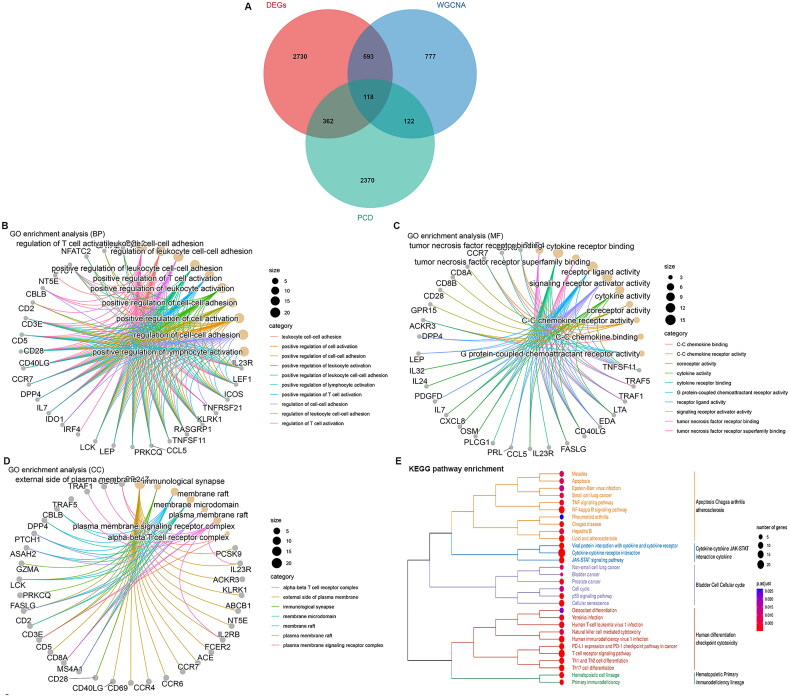
Functional analysis of key genes. A, Key genes screened by Venn analysis. B, GO enrichment analysis of key genes in BP (biological process) category. C, GO enrichment analysis of key genes in MF (molecule function) category. D, GO enrichment analysis of key genes in CC (cellular component). E, Tree diagram of significant KEGG pathways for key genes.

In total, 118 key genes were identified by overlapping PCD-associated genes with DEGs and significant WGCNA module genes (Figure 3(A)). Function enrichment analysis suggests that the overlapped genes were closely related with the immune cell regulation in BP category, such as regulation of T-cell activation, leukocyte cell-cell adhesion, and regulation of leukocyte cell-cell adhesion (Figure 3(B)). For MF category, genes were significantly enriched in tumour necrosis factor receptor binding, cytokine receptor binding and tumour necrosis factor receptor superfamily binding (Figure 3(C)). For CC, genes were closely related with external side of plasma membrane and immunological synapse (Figure 3(D)). The significant pathways enriched by key genes are mainly associated with apoptosis, cell cycle, and cytokine–cytokine interaction (Figure 3(E)).

### PPI network

**Figure 4.**
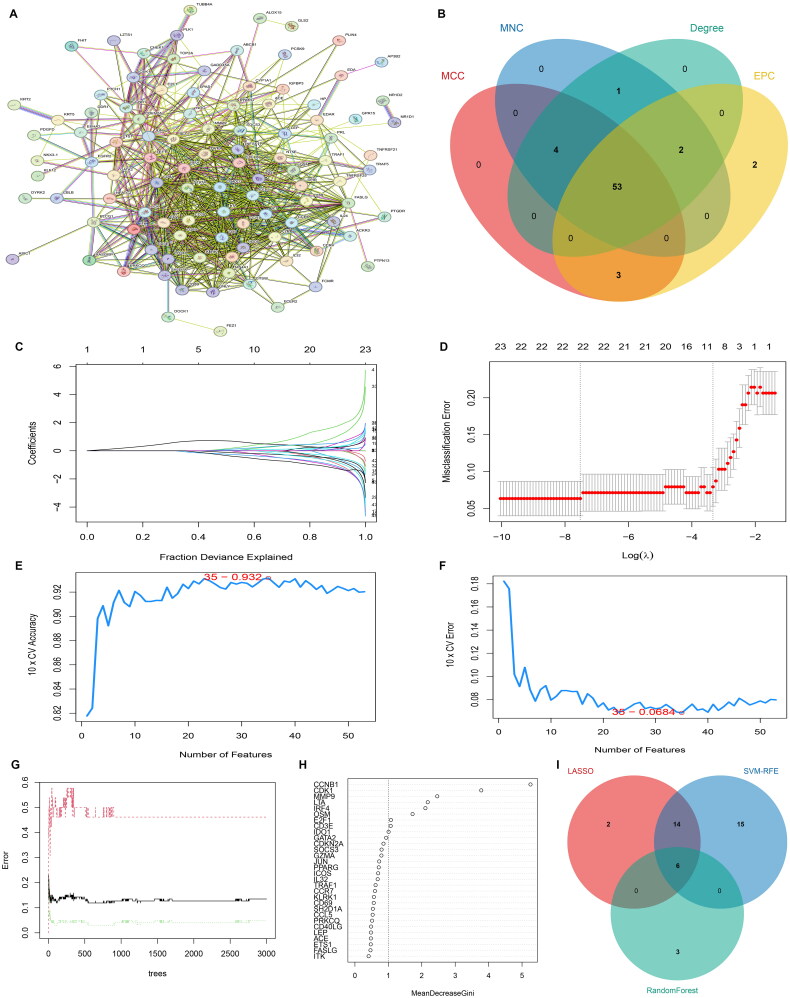
Identification of biomarkers of COVID-19. A, protein-protein interaction (PPI) network. B, Venn diagram of the PPI hub genes based on four topology analysis algorithms (MCC, MNC, EPC and Degree). C and D, LASSO algorithm for characteristic genes selection. E and F, Characteristic genes selection via SVM-RFE algorithm. G and H, Characteristic genes selection via random forest algorithm. I, Venn diagram of biomarkers based on three machine learning methods.

The PPI network was constructed using String and Cytoscape software with 103 nodes and 780 edges (Figure 4(A)). Totally, 53 common hub genes were screened based on four methods: MCC, MNC, EPC and Degree (Figure 4(B)).

### Feature genes screened by machine learning methods

To filter out feature genes from 53 hub genes, three regression models were carried out. LASSO regression model was constructed and identified 22 genes through 10-fold cross-validation (Figure 4(C,D)). For SVM-RFE method, 35 feature genes were screened out (Figure 4(E,F)). In addition, nine feature genes were screened out by RF algorithm (Figure 4(G,H)). Finally, only six overlapping feature genes (CCNB1, CDK1, IRF4, LTA, MMP9 and OSM) were filtered out as biomarkers (Figure 4(I)).

### The expression and performance evaluation of biomarker genes

**Figure 5.**
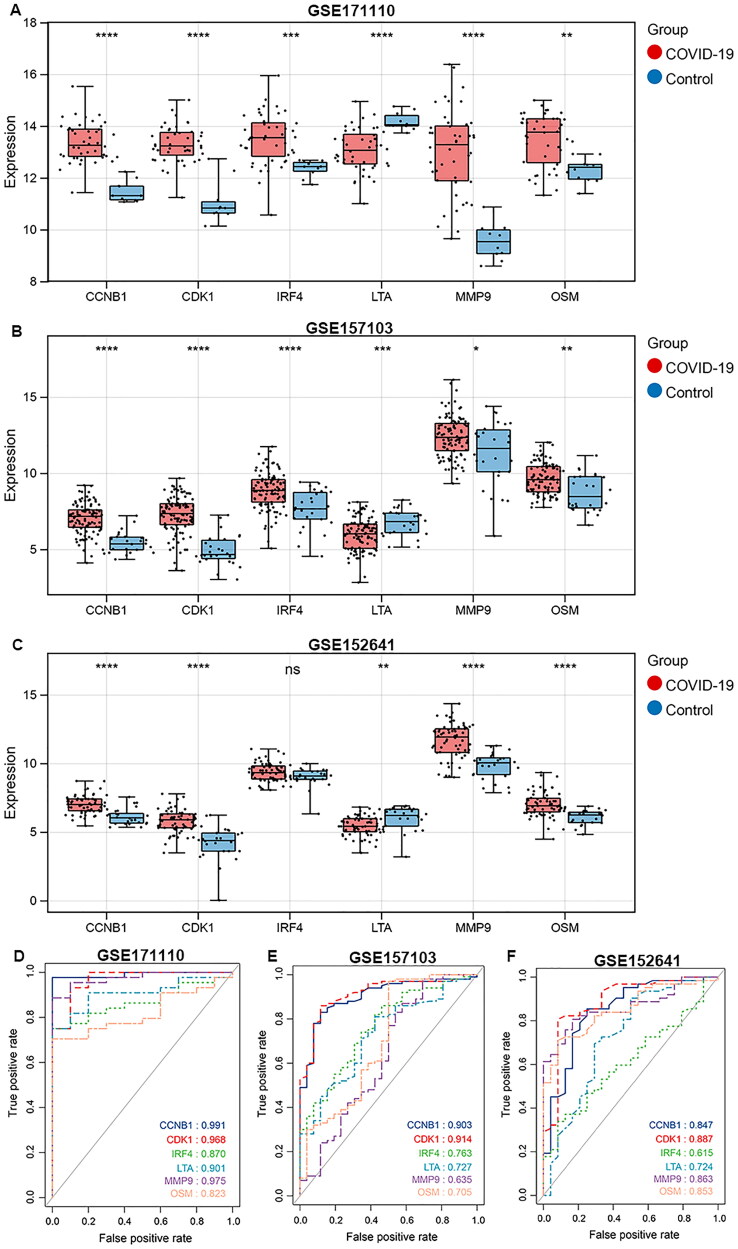
Expression profiles and diagnosis performance of biomarkers. A, Box plot of gene expression patterns of biomarkers in GSE171110 training dataset. B, GSE157103 validation dataset. C, GSE152641 validation dataset. D, ROC curve analysis of the diagnostic performance of biomarkers in GSE171110 training dataset. E, GSE157103 validation dataset. F, GSE152641 validation dataset.

The expressions of CCNB1, CDK1, IRF4, MMP9 and OSM were significantly higher in COVID-19 samples compared with matched controls, while LTA was down-regulated in GSE171110 training dataset (Figure 5(A)). The similar expression profile of six biomarkers was validated in GSE157103 and GSE152641 datasets (Figure 5(B,C)). To evaluate the diagnostic performance of the biomarkers in COVID-19, ROC curve analyses were performed. In GSE171110 training dataset, all the six biomarker genes elicited strong diagnostic capabilities with area under the curve (AUC) greater than 0.8 (Figure 5(D)). In GSE157103 dataset, AUCs of all the biomarkers were greater than 0.635, with the highest AUC value of 0.914 for CDK1, and 0.903 for CCNB1 (Figure 5(E)). In GSE152641, CCNB1 achieved an AUC of 0.847, CDK1 had an AUC of 0.887, IRF4 had an AUC of 0.615, LTA had an AUC of 0.724, MMP9 had an AUC of 0.863 and OSM had an AUC of 0.853 (Figure 5(F)).

### Correlation between biomarkers with clinical information

**Figure 6.**
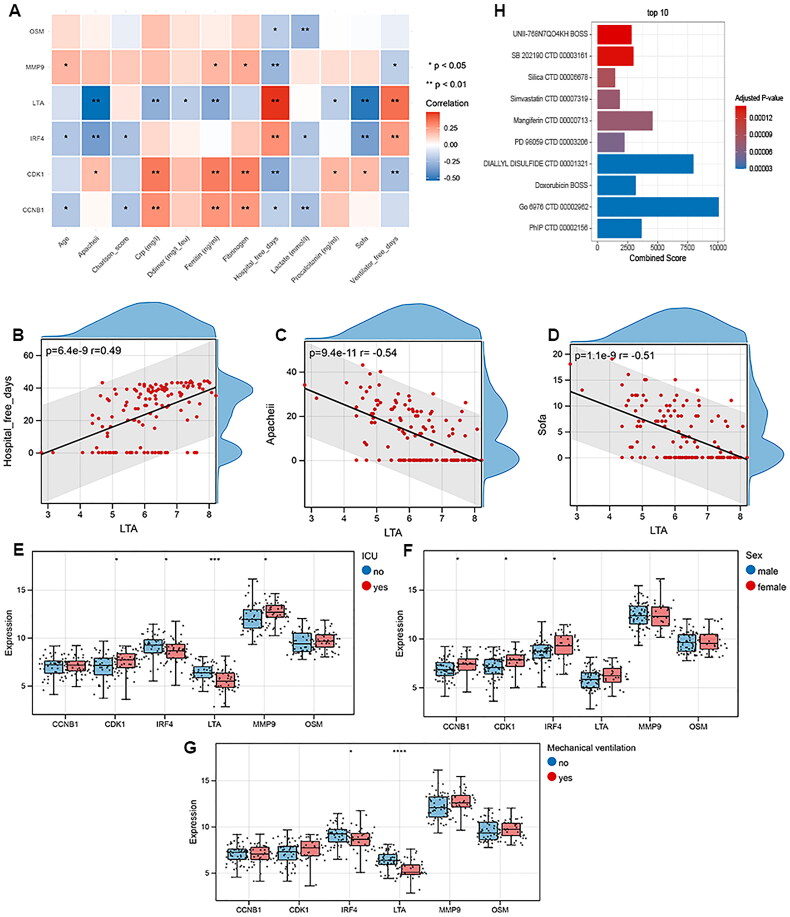
Correlation between biomarkers with clinical characteristics. A, Correlation matrix of the biomarkers with clinical indicators. Correlations are analyzed by Pearson correlation. B, Linear correlation between LTA with hospital-free days. C, Correlation between LTA with apacheii. D, Correlation between LTA with SOFA scores. E, Box plot of the expression of biomarkers between patients with intensive care unit (ICU) stays and those without ICU admission. F, Box plot of the biomarker expression in male patients and female patients. G, biomarker expression in patients with and without mechanical ventilation. *p < 0.05; **p < 0.01, ***p < 0.001, ****p < 0.0001. H, top 10 potential drugs targeting biomarkers.

To assess the association between biomarker genes with clinical characteristics, Pearson correlation analysis was performed. As depicted in Figure 6(A), the biomarker genes exert diverse associations with clinical indicators. LTA shows positive correlation with hospital-free days, while negative correlation with Apacheii and Sofa (Figure 6(B–D)). In the training dataset, the expressions of CDK1, IRF4, MMP9 and LTA are significantly different between patients with and without intensive care unit (ICU) stays (Figure 6(E)). CCNB1, CDK1 and IRF4 are higher expressed in female patients, compared with male ones (Figure 6(F)). In addition, IRF4 and LTA were differentially expressed in patients with and without mechanical ventilation (Figure 6(G)).

### Drug predictive analysis

The potential drugs targeting six biomarker genes were retrieved from DSigDB database. The top 10 drugs ranking by combined scores are shown in Figure 6(H), such as simvastatin, mangferin, and doxonrubicin.

### Nomogram model construction

**Figure 7.**
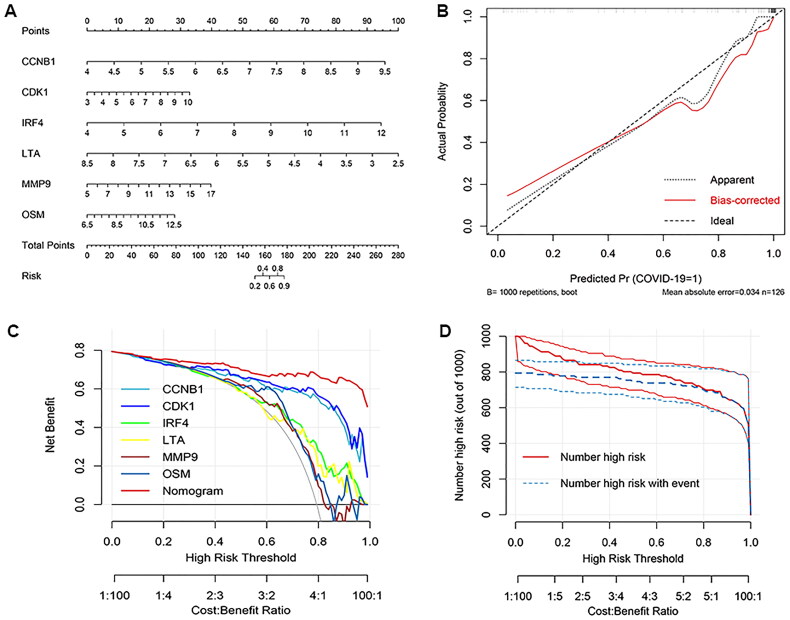
Diagnostic nomogram model construction based on 6 biomarker genes. A, diagnostic nomogram model for COVID-19. B, Calibration curve of the diagnostic nomogram model. C, Decision curve analysis (DCA) for clinical significance of the nomogram model. D, Clinical impact curve of the nomogram model.

To validate the diagnostic performance of six biomarker genes for COVID-19, we constructed nomogram model (Figure 7(A)). The predictive accuracy of nomogram model was determined by calibration curve. As illustrated in Figure 7(B), the actual predictive curve is similar with the corrected curve, indicating a high prediction value of nomogram. The decision curve analysis (DCA) curve shows that nomogram had a better net benefit than single biomarker (Figure 7(C)). In clinical impact curve, the predicted probability of patients with high risk of COVID-19 is close to the actual probability as the risk threshold from 0.6 to 1 (Figure 7(D)).

### Immune cell infiltration

**Figure 8.**
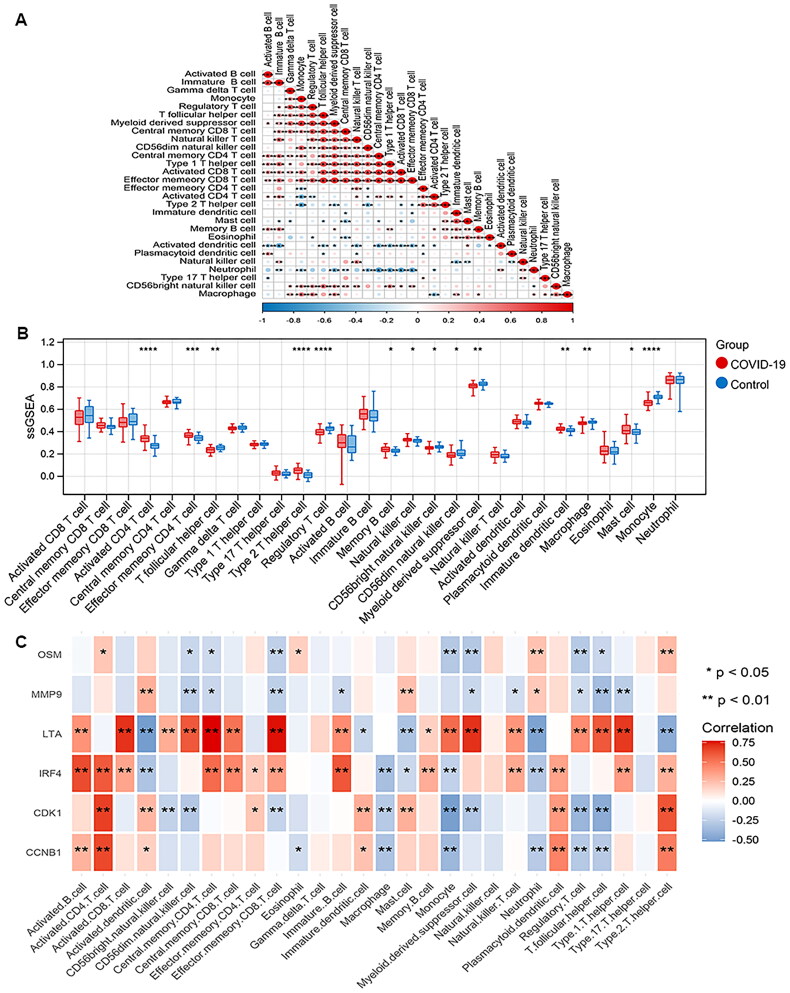
Immune microenvironment in COVID-19. A, Heatmap of the correlation between immune cells. *p < 0.05; **p < 0.01, ***p < 0.001, ****p < 0.0001. B, Comparison of the immune cell infiltration between COVID-19 and healthy controls. C, Correlation between biomarkers with immune cells.

Based on GSE171110 dataset, the abundance of 28 types of immune cells infiltration was quantified using ssGSEA algorithm. The correlations between immune cells were analysed by Pearson correlation analysis (Figure 8(A)). There are 14 types of immune cells with differential infiltration between COVID-19 and control samples, such as activated CD4 T cell, memory effector CD4 T cell and follicular helper T cell (Figure 8(B)). The six biomarker genes showed various correlations with immune cells (Figure 8(C)).

### Clusters identification in COVID-19 patients

**Figure 9.**
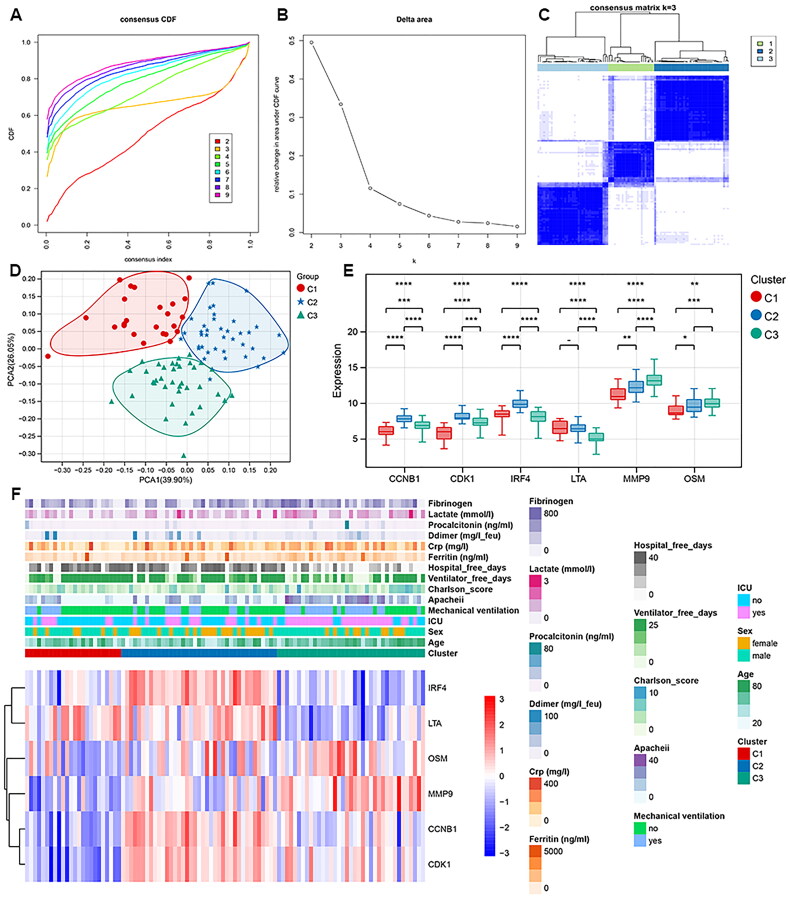
Subtypes analysis in COVID-19 patients. A, Consensus CDF plot. B, Consensus CDF plot. C, Consensus matrix heat map. D, PCA for the clusters. E, Box plot analysis of the biomarker genes in different clusters. F, heatmap of the expression profiles of biomarker genes.

Six biomarker genes were used to cluster COVID-19 patients. The consensus distribution of clusters is depicted in cumulative distribution function (CDF) curve (Figure 9(A)). The changes in area under CDF curve were determined with k from 2 to 9. There was a great changes of areas between *k* = 3 and *k* = 4 and after which the areas changed substantially (Figure 9(B)). *K* = 3 was found to be the optimal cluster count with distinct clusters of samples (Figure 9(C)). PCA showed that the samples in cluster 1 (*n* = 24), 2 (*n* = 39), 3 (*n* = 37) were distinguished clearly (Figure 9(D)). The six biomarker genes exerted differential expression between different clusters (Figure 9(E)). The heatmap of differential expression profiles of six biomarker genes in cluster 1, 2, 3 is depicted in Figure 9(F).

### Correlation between clusters with clinical information and immune microenvironment

**Figure 10.**
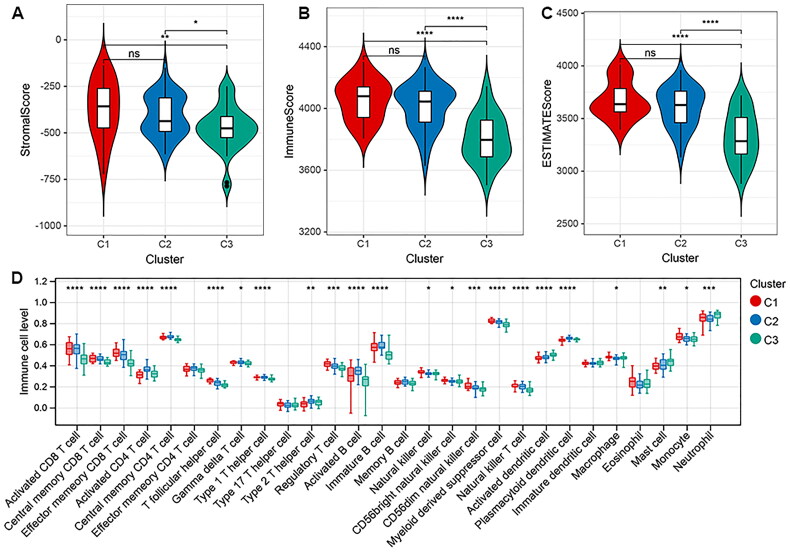
Comparison of immune microenvironment in different clusters. A, Stromal score analysis. B, Immune score. C, ESTIMATE score. D, Comparison of the immune cell infiltration in different clusters based on ssGSEA method. *p < 0.05; **p < 0.01, ***p < 0.001, ****p < 0.0001.

As shown in Table 2, COVID-19 clusters are significantly correlated with multiple clinical features, such as sex, age, and hospital-free days (*p* < 0.05). To compare the microenvironment between clusters, the stromal score, immune score, and estimate score were calculated based on estimate algorithm. The results showed that there were significant different stromal score, immune score, and estimate score between cluster 1 and cluster 3, cluster 2 and cluster 3 (all *p* < 0.05, Figure 10(A–C)). Totally, 23 of 28 types of immune cells showed differential infiltration based on clustering (Figure 10(D)).

**Table 2.** Correlation between clusters with clinical indicators.

Characteristics	C1 (*N* = 24)	C2 (*N* = 39)	C3 (*N* = 37)	Total (*N* = 100)	*P* value
Sex					0.03
female	6(6.00%)	21(21.00%)	11(11.00%)	38(38.00%)	
male	18(18.00%)	18(18.00%)	26(26.00%)	62(62.00%)	
ICU					<0.001
no	17(17.00%)	24(24.00%)	9(9.00%)	50(50.00%)	
yes	7(7.00%)	15(15.00%)	28(28.00%)	50(50.00%)	
Mechanical ventilation					0.02
no	16(16.00%)	27(27.00%)	15(15.00%)	58(58.00%)	
yes	8(8.00%)	12(12.00%)	22(22.00%)	42(42.00%)	
Age					0.01
Mean ± SD	60.25 ± 16.95	55.28 ± 17.45	66.73 ± 14.03	60.71 ± 16.74	
Median[min-max]	62.00[27.00,87.00]	55.00[20.00,86.00]	68.00[33.00,89.00]	62.00[20.00,89.00]	
Apacheii					<0.001
Mean ± SD	6.79 ± 8.95	8.41 ± 10.79	19.62 ± 12.21	12.17 ± 12.28	
Median[min-max]	0[0,27.00]	0[0,31.00]	21.00[0,43.00]	12.00[0,43.00]	
Charlson_score					0.13
Mean ± SD	3.00 ± 2.00	2.82 ± 2.44	3.95 ± 2.71	3.28 ± 2.48	
Median[min-max]	2.50[0,7.00]	2.00[0,9.00]	3.00[0,11.00]	3.00[0,11.00]	
Ventilator_free_days					0.01
Mean ± SD	23.79 ± 9.36	21.51 ± 10.68	15.43 ± 12.56	19.81 ± 11.56	
Median[min-max]	28.00[0,28.00]	28.00[0,28.00]	20.00[0,28.00]	28.00[0,28.00]	
Hospital_free_days					<0.001
Mean ± SD	27.54 ± 16.03	29.10 ± 14.83	11.16 ± 12.95	22.09 ± 16.62	
Median[min-max]	32.00[0,44.00]	35.00[0,43.00]	3.00[0,39.00]	26.00[0,44.00]	
Ferritin (ng/mL)					<0.001
Mean ± SD	636.21 ± 1207.61	809.31 ± 1121.09	1103.97 ± 934.57	876.79 ± 1083.49	
Median[min-max]	283.00[0,5971.00]	401.00[32.00,5508.00]	1035.00[0,4609.00]	592.50[0,5971.00]	
Crp (mg/l)					0.04
Mean ± SD	96.44 ± 119.67	128.48 ± 98.79	151.46 ± 102.50	129.30 ± 106.48	
Median[min-max]	53.00[0,430.50]	124.20[0,408.80]	133.60[0,350.60]	122.50[0,430.50]	
Ddimer (mg/l_feu)					0.05
Mean ± SD	10.91 ± 28.91	11.11 ± 23.19	8.14 ± 11.25	9.96 ± 21.18	
Median[min-max]	0.89[0,104.42]	1.28[0,102.21]	2.21[0 + 0,51.70]	1.54[0,104.42]	
Procalcitonin (ng/mL)					0.04
Mean ± SD	2.29 ± 7.30	1.39 ± 3.47	4.67 ± 14.54	2.82 ± 9.80	
Median[min-max]	0.20[0,36.00]	0.25[0,18.00]	0.98[0,86.39]	0.46[0,86.39]	
Lactate (mmol/l)					0.1
Mean ± SD	0.56 ± 0.58	0.75 ± 0.75	0.96 ± 0.75	0.78 ± 0.72	
Median[min-max]	0.54[0,1.53]	0.86[0,2.85]	1.01[0,3.28]	0.86[0,3.28]	
Fibrinogen					0.25
Mean ± SD	369.38 ± 274.91	411.74 ± 288.41	487.59 ± 279.34	429.64 ± 283.06	
Median[min-max]	400.00[0,929.00]	479.00[0,910.00]	489.00[0,949.00]	473.00[0,949.00]	

### Clinical sample verification by RT-qPCR assay

**Figure 11.**
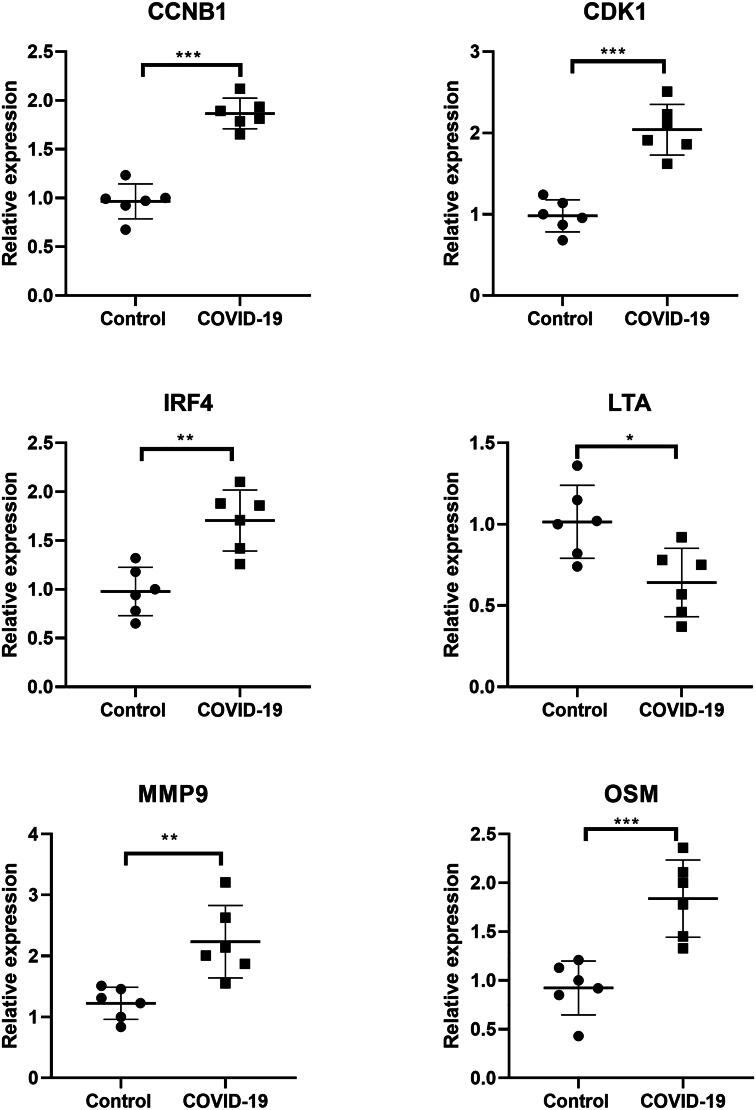
RT-qPCR verification. The differential expressions of six biomarker genes were verified in 6 paired blood samples from COVID-19 and healthy controls. *p < 0.05, **p < 0.01, ***p < 0.001.

The differential expressions of six biomarker genes were verified by real time PCR assay. Consistent with the bioinformatic analysis, CCNB1, CDK1, IRF4, MMP9 and OSM were significantly overexpressed, while LTA was de-expressed in COVID-19 patients compared with healthy controls (Figure 11).

## Discussion

Effective biomarkers may facilitate the stratification of patients with high or low risk of disease and help for treatment option and complication prevention. Currently, many biomarkers reflecting the major pathophysiological features of the disease have been identified and correlated with the risk of developing severe disease [27]. Although blood-based biomarkers have been widely predicted for the diagnosis of COVID-19, no robust and reliable biomarkers are available in clinical practice currently. Compelling evidence has determined the critical role of PCD in the progression of COVID-19 [6,28]. RNA-seq allows comprehensive transcriptome analysis and machine learning approaches are helpful for the discovery and validation of biomarkers. Thus, in this study, we attempted to discover potential biomarkers related with PCD for the risk stratification of COVID-19 patients and effective prevention of severe complications based on RNA-seq data by utilizing machine learning methods.

COVID-19 is a severe respiratory disease associated with various short-term and long-term complications, such as cardiovascular, neurological and pulmonary injuries [29]. A large number of patients are presented from asymptomatic to multi-organ dysfunction, and even death [30]. The reliable biomarkers would be helpful for risk stratification and facilitate early diagnosis and intervention for COVID-19. To screen the candidate blood-based biomarkers, three gene expression datasets of the blood samples from COVID-19 and matched controls were downloaded and performed comprehensive bioinformatic analysis. We investigated the DEGs in COVID-19 compared with matched healthy controls. The clinical relevant and PCD associated DEGs were significantly enriched in apoptosis pathways. Apoptosis also served as a PCD, which has been found to be dysregulated in various diseases, including bacteria and virus infectious diseases. TNF signaling pathway was significantly enriched by DEGs. It has been reported that TNF α, served as pro-inflammatory cytokine, plays a key role in mediating inflammatory response, apoptosis and proliferation [31]. The serum level of TNF α is elevated and exacerbates inflammatory response in COVID-19. TNF α is a biomarker for the severity and prognosis of COVID-19 [32]. TNF signalling has been suggested as the therapeutic target for COVID-19 [33]. These suggested that PCD was dysregulated in COVID-19 and these genes are critical in the pathogenesis of COVID-19.

Next, the biomarker mining was achieved by utilizing PPI network and machine learning methods. Finally, six potential biomarkers were screened, including CCNB1, CDK1, IRF4, LTA, MMP9 and OSM. CCNB1 (Cyclin B1) is a mitosis-related protein, which is involved in cell cycle by controlling G2/M transition. The reduced levels of CCNB1 is associated with the inhibition of anti-apoptosic Bcl-2 protein mediated by obatoclax (OLX) and B cell lymphoma-2 (Bcl-2) inhibitor ABT-737. Bcl-2 inhibitors exert potent antiviral activities against COVID-19 [34]. CDK1 is a cyclin-dependent kinase, which exerts essential role in cell cycle. The CDK inhibitor is reported to show a strong anti-viral activity and exert an efficacy in inhibiting severe acute respiratory syndrome coronavirus 2 (SARS-CoV-2) replication [35]. A previous observational study has suggested that CCNB1 and CDK1 is the hub regulatory genes in the pathogenesis of COVID-19 and highly expressed in COVID-19 [36]. Their excellent diagnostic values were determined by ROC curve analysis, with AUC values larger than 0.9 [36]. These findings were consistent with our results. The abnormal expression of CCNB1 and CDK1 indicated that the cell cycle and proliferation was dysregulated in COVID-19.

Further, OSM is a pleiotropic cytokine belonging to interleukin-6 family. OSM is implicated in various inflammatory response and a broad spectrum of inflammatory diseases [37]. Mozaffarian et al. have reported that OSM plays a key role in the development and progression of lung diseases [38]. The elevated OSM signalling in lung inflammatory diseases can be served as the therapeutic target [37]. COVID-19 infection is closely linked with severe inflammatory responses [39]. Our data determined the overexpression of OSM in COVID-19 patients, indicating the potential of OSM as the new target for COVID-19. In our study, the ROC curve suggested the AUCs of the biomarkers were larger than 0.8 in training dataset and above 0.6 in validation datasets. The dysregulation of the six feature genes may be served as the biomarker for early diagnosis of COVID-19.

Previous evidence has indicated that the epidemiological characteristics of COVID-19 were different with regard to gender [40]. Males have higher susceptibility to COVID-19 infection [41]. Males with COVID-19 infection are inclined to be associated with a higher severity, higher need of ICU and worse outcomes than females [10]. Our data further revealed that CCNB1, CDK1 and IRF4 showed sex-based expression, while IRF4 and LTA showed differential expression in patients with and without ICU admission. These implied that the sex- and ICU-based expression of biomarkers could predict disease severity, ICU admission and mortality.

Our final model suggested that COVID-19 patients could be classified into three subtypes. The three clusters showed differential clinical features, including age, sex and ICU admission. The immune scores and 23 types of immune cell levels were also different between three clusters. It is reported that the level of CD8 T cells was significantly different in COVID-19 patients compared with matched controls, which triggered the immune response after COVID-19 infection [10]. Macrophages, as the innate immune cells is reported to contribute to the progression of COVID-19, by producing a large amount of interleukin 6 (IL-6) [42]. Monocytes are recruited after viral infections and play roles in the defense of pathogens. The pulmonary macrophages derived from inflammatory monocytes are excessively activated after COVID-19 infection, resulting in inflammatory cytokines release and tissue damage exacerbation [43]. Our data showed that the six biomarkers were differentially expressed in three clusters. Collectively, the biomarkers related with clinical indicators and immune response can stratify COVID-19 patients, which may facilitate personalized treatment in patients.

Although we have achieved relatively satisfactory results. However, several limitations cannot be ignored. Firstly, fewer samples were collected in the validation experiment, which may affect the accuracy of the results. Second, the efficacy of the vaccine is beyond doubt, vaccination can limit the occurrence of the disease, and the antibody protection generated should be considered as a variable in the statistical evaluation [44], the factor of vaccination history was not taken into account in this analysis. Next, we will design the experiment better and expand the sample size to further verify our conclusions.

## Conclusion

In conclusion, we employed bioinformatic tools and machine learning method to explore the biomarkers for COVID-19. Six PCD-related genes were identified to be feature genes in COVID-19, which were differentially expressed in COVID-19 compared with matched controls. The biomarkers showed excellent or good diagnostic value and could classify patients with different risks of COVID-19. These genes were correlated with clinical indicators and immune cell infiltration, which could be used as the biomarkers for the treatment for COVID-19, and guide the early intervention of similar disease.

## Data Availability

The data presented in this study are available on request from the corresponding author.
